# Cyclopædia of Anatomy and Physiology

**Published:** 1845-07

**Authors:** 


### MEDICO-CHIRURGICAL

### REVIEW.

JULY, 1845.

I. The Cyclopædia of Anatomy and Physiology. Parts 25 and 26. 1844. Articles:—Nerve, Dr. *Todd;* Nervous System, (Comparative Anatomy), by *J. Anderson,* Esq. Nervous Centres, Dr. *Todd.*

II. The Physiological Anatomy and Physiology of Man. By *R. B. Todd,* M.D., F.R.S., and *William Bowman,* F.R.S. Part II. 1845.

III. On the Nervous and Circulating Systems in Myriapoda and Macrourous Arachnida. By *G. Newport,* F.R.S. Philosophical Transactions. 1843.

IV. Ueber den Verlauf der Nervenfasern im Rückenmarke des Frosches von Dr. *Julius Budge.* Erfahrungen über die Functionelle Selbständigkeit des Sympathischen Nervensystems, von *F. Bidder.* Vorläufige Mittheilung über die Structur der Ganglien und den Ursprung der Nerven bei wirbellosen Thieren. Von Dr. *Friedrich Will.* Neurologische Erläuterungen, von Dr. *R. Remak.* Muller's Archiv. 1844.

V. Lehrbuch der Physiol. des Menschen. Band. II. Von. Dr. *G. Valentin.* 1844.

VI. Recher. Experim. sur les Fonctions du Nerf Spinal. Par *C. Bernard.* Archiv. Gen. de Med. 1844.

It must be confessed that, although within the last few years there has been no lack of labourers in the field of neurology, the actual progress has not been commensurate with all the toil and trouble that has been bestowed upon the subject. In some of the more recent contributions indeed, and herein our friends of the German school have been the principal offenders, the tendency has been to retrograde. We certainly did not anticipate, and least of all in England, that a time would arrive when it would be necessary to vindicate the great principle announced by Bell, that principle upon which *all* accurate knowledge of the nervous system must ever repose, the individuality, namely, and uninterrupted continuity of the primary nervous filaments. Doubts have, however, been thrown upon this fundamental truth; and principally, as it would appear, on the faith of certain dissections and experiments of Stilling, from which it is inferred that the elementary fibres interlace and are fused one with another in the spinal cord, in the several nervous plexuses, and in other parts of the system; that, in fact, the disposition of the nervous system "is precisely analogous to what is seen in the venous and lymphatic systems."* We believe this to be an entire fallacy, and unhesitatingly express our conviction, resting on repeated and careful examination, that no anatomist has *ever seen*, in any of the parts just mentioned, a true anastomosis in the sense of that existing in the vascular system. The only places where the primary nervous tubules do actually communicate is in their peripheral extremities, where, both in the muscles and in the tactile papillæ of the skin, they unite and form loops. It has also been asserted by Valentin, that a similar disposition prevails in the central ends, implanted in the grey matter of the brain, but, as we shall subsequently state, this must at present be held to be doubtful.

It is so essential to the physiology and pathology of the nervous system to establish the real relations of its component parts, that we conceive the best introduction to the subject before us, will consist of a brief reference to the disposition of the nervous tubules. And, in the first place, it may not be superfluous to remark that the functions of the blood-channels and nerves are so totally different, that it is surprising any comparison between them should have been attempted. It is the office of the blood-vessels not merely to carry the nutritive fluids to all parts of the body, but specially, by overcoming the repeated obstacles which impede the circulation, to secure, in the extreme divisions or capillaries, that uniform current which alone is compatible with healthy nutrition. Now, how is this to be accomplished?—clearly by providing free inter-communications between all the parts of the vascular system, and more particularly between its smaller divisions. Thus, as to the function of the blood-vessels, it matters not *how* the blood reaches its destination, provided it does but get there. But in the case of the nervous system all is reversed; here the whole action of innervation requires in theory what is shown by observation, a disposition of the nervous threads, which will enable them to act as isolated conductors, so as to transmit unmingled the mandates of the will centrifugally to special muscles, and centripetally the impressions made on the organs of sense to the brain.

In consequence of the unsatisfactory results of many of Stilling's researches, we have again cautiously examined the disposition of the primitive tubes in the spinal cord, with the express object of ascertaining if, in any part of the white substance, an anastomosis could be detected. All our examinations have shown that the fibres *invariably observe an isolated course*. When viewed with a sufficiently high power, an objective of one-sixth of an inch focus for example, the beaded particles are beautifully and distinctly seen in a perfectly recent specimen, and, mixed with them, branches of minute blood-vessels and capillaries; these latter canals, it is necessary to state, present appearances so deceptive, that to an unpractised eye, and especially when low powers are used, as was done by Stilling, they may very readily be mistaken for nervous tubes, and herein we believe will be found the source of the errors above noticed. The capillary vessels are seen branching and uniting, but they present physical

* British and Foreign Medical Review, 1844, p. 146.

marks, and especially nuclei, in their walls, which are quite distinctive: indeed we were never more impressed with the certainty and elucidation which microscopic examination confers on the great questions of physiology, than by thus seeing under the eye the true characteristics of the vascular and nervous systems.

The conclusion at which we have ourselves arrived, as the result of direct inspection, is confirmed by the best observers. Thus Valentin, in his Physiology just completed, after pointing out and demonstrating the physiological necessity of isolated conductors in the peripheral portion of the nervous system, both as relates to sensation and motion, says, "We conclude, as a general anatomical proposition, that all ramifications, anastomoses, and interlacements of the nerves, are in reality only apparent, and therefore that no true divisions nor communications similar to those of the blood-vessels exist, but merely a corresponding entrance or exit of unbranched, uninterrupted primitive fibrils; a disposition which enables us easily to comprehend the laws of nervous conduction."—(*Lehrbuch der Physiol. Zweiten Band.*, *p.* 589.)

In the same sense Dr. Todd, in his excellent article upon the nervous system (*Cyclop. of Anat. and Phys.*, *Part* 25, *p.* 593), thus expresses himself: "the nerve-tubes lie side by side, parallel, and sometimes have a wavy course within the general sheath. The relation of the nerve-tubes to each other is simply that of juxta-position. All observers, from Fontana down to those of the present day, agree in denying the existence of any inosculation or anastomosis between the fibres in vertebrate animals; and it seems almost certain that this complete isolation of the nerve-tubes is not limited to those of the nerves, properly so-called, but may be observed in the nervous centres also." A similar opinion is expressed by the same gentleman in the recent number of the Physiological Anatomy.

Although there is no real anastomosis, all the nerves, with the exceptions probably of the olfactory, optic, and perhaps of the acoustic, mutually interchange fibres, sentient nerves receiving motor fibrils, and muscular nerves receiving sentient twigs, by which their actions are modified in a most important manner.

In the preceding observations, there is expressed what we are convinced is the law which regulates the course of the nerve-tubes; and we only further remark that, if anatomists yield this fundamental fact in obedience to some recent, and in many respects defective, researches, they must at the same time be prepared to abandon that which is the key to all Bell's splendid discoveries, and to return to the chaos of confusion, uncertainty, and contradiction from which this, the highest, branch of physiological science was rescued by our illustrious countryman.

In considering the works and papers before us, we shall commence with the

Structure of the Gray and Fibrous Substances.—Although organic chemistry is not sufficiently advanced to throw much light on the nervous system, it is interesting to know that, according to L'Heritié, the quantity of phosphorus found in the nervous matter varies considerably at different periods of life, and that it is very small proportionally in idiotcy. The following table of his comparative analyses, is taken from the Physiological Anatomy of Dr. Todd and Mr. Bowman.

| | Infants. | Youth. | Adults. | Old Men. | Idiots. |
|---|---|---|---|---|---|
| " Albumen | 7·00 | 10·20 | 9·40 | 8·65 | 8·40 |
| Cerebral fat | 3·45 | 5·30 | 6·10 | 4·32 | 5·00 |
| Phosphorus | 0·80 | 1·65 | 1·80 | 1·00 | 0·85 |
| Osmazome and Salts | 5·96 | 8·59 | 10·19 | 12·18 | 14·82 |
| Water | 82·79 | 74·26 | 72·51 | 73·85 | 70·93 |
| | 100·00 | 100·00 | 100·00 | 100·00 | 100·00." |

VESICULAR OR CORPUSCULAR NERVOUS MATTER.—This consists of a fine granular matter, in which are imbedded the well-known ganglionic corpuscles or nerve-vesicles. In the Physiological Anatomy the gray substance is thus described.

" The essential elements of the gray nervous matter are *vesicles* or cells, containing nuclei and nucleoli. They have been also called *nerve* or *ganglion globules*. The wall of each vesicle consists of an exceedingly delicate membrane, containing a soft but tenacious finely granular mass. The *nucleus* of the cell is generally eccentric, much smaller than the containing vesicle, and adherent to some part of its interior. Its structure is apparently the same as that of the outer vesicle. The *nucleolus* is a minute, remarkably clear, and brilliant body, also vesicular, inclosed within the nucleus. It forms a most characteristic and often conspicuous part of the nerve-vesicle. The ordinary or prevailing form of these elements is that of a globular vesicle. So soft and compressible are they, however, that a good deal of diversity of shape is manifest in them, by reason of the compression they suffer as they lie packed together *in situ*. Hence some are spherical, others ovoidal, or ellipsoidal. In some vesicles we find, external to the nucleus, particles of a coarser kind, which are accumulated in a mass, frequently of a semilunar form. These are pigment granules; their presence gives a dark colour to a portion of the vesicle. Sometimes we find two groups of pigment granules in one vesicle. They are usually of a reddish or yellowish brown colour." P. 213.

The work just quoted also gives a more satisfactory explanation of what are called " caudate " vesicles or corpuscles than has hitherto appeared.

" Another form of nerve-vesicle is characterized by one or more tail-like processes extended from it, and to such nerve-vesicles we may apply the term *caudate*. They possess the nucleus and nucleolus, as in the more simple form; and contain one or more masses of pigment, which are often of very considerable size. Both the vesicles and their caudate processes vary greatly in size and shape. The largest nerve-vesicles are found among those of this kind. Sometimes there is but a single process from a vesicle; or there may be two, proceeding from opposite sides; or there may be several, extending in various directions. There is great difference in the shape of these caudate vesicles, as may be observed in figs. 55 and 56, where different varieties of them have been represented. In point of structure, the caudate processes are exceedingly delicate, and finely granular, like the interior of the vesicle, with which they distinctly seem to be continuous. Such is the delicacy of these processes, that they readily break off; in general, very close to the vesicle. Sometimes, however, one or more of them may be traced to a considerable distance, and will be found to divide into two or into three branches, which undergo a further subdivision, and give off some extremely fine transparent fibres, the connexion of which with the other elements of the nervous tissue has yet to be ascertained. It is most probable, however, that they either serve to connect distant vesicles, or else that they become continuous with the axis cylinders of the tubular fibres. In the cerebro-spinal centre, we have found the tissue in the vicinity of the caudate vesicles freely traversed in all directions by numerous very delicate filaments, which seem to be the ramifications of the caudate processes. These often exhibit considerable tenacity and elasticity." P. 214.

If the connexion between these vesicular prolongations and the nervous tubules should be proved, it would be one step gained towards the explanation of the connexion existing between the organ—the gray substance which generates the nervous force, and the instruments, the tubules, which serve to transmit its effects.

Of the Fibrous Nervous Matter.—This forms by far the larger portion of the substance of the nervous system, constituting not only almost the entire mass of the nerves and the whole of the so-called medullary matter of the spinal cord and encephalon, but also a very considerable proportion of the gray substance in every situation—in the convolutions of the brain, in the laminæ of the cerebellum, in the interior masses, such as the striated bodies, thalami and so forth, in the axis of the spinal cord and in all the ganglions. We extract the following account of this structure, embodying all that is at present known respecting it, from the work quoted above.

"*Of the Tubular Fibre.*—When a nerve-tube is perfectly recent, and unaffected by re-agents, it presents, if viewed by reflected light, a beautifully pearly lustre, and appears to be quite homogeneous. But if viewed by transmitted light, and with a sufficient magnifying power, a more complicated structure becomes visible in all the largest and best marked specimens. Most externally is the *tubular membrane*, an homogeneous, and probably elastic tissue of extreme delicacy, analogous to the sarcolemma of striped muscle, and, according to our observation, not presenting any such distinct, longitudinal, or oblique fibres in its composition as have been described by some writers. Within the edge of the tubular membrane, on each side, are seen two thicker and darker lines, which appear to mark the outer and inner limits of an inner layer of different composition and refracting power, and which is generally known as the *white substance of Schwann.* This forms a tube within the tubular membrane. Within the white substance of Schwann is a transparent material, occupying the axis of the nerve-tube. This has been called by Remak the *flattened Band;* but a better name for it is that of *axis cylinder*, employed by Rosenthal and Purkinje. It is evident, that the whole of the matter contained in the tubular membrane is extremely soft, for it is found to yield under very slight pressure, and may be readily made to pass from one part of the tube to another." P. 209.

The tubular fibres vary in diameter from $\frac{1}{1600}$ to even $\frac{1}{10000}$ of an inch, but the average is from $\frac{1}{2000}$ to $\frac{1}{4000}$ of an inch.

Dr. Todd and Mr. Bowman admit the existence of an independent gelatinous and *solid* nerve-fibre, found principally in the sympathetic system. As there is no point connected with the structural anatomy of the nervous system on which more contradictory opinions prevail, no apology will be required for laying before our readers the latest, and we may add the best, account of this fibre.

"*Of the Gelatinous Nerve-fibre.*—This term is applied by Henle to certain fibres found principally in the sympathetic nerve. They are flattened, soft, and homogeneous in appearance; containing numerous cell-nuclei, some of which are round, others oval; some situated in the centre of the fibre, others adhering to either edge; their longest diameter being generally parallel to the longitudinal axis of the nerve. These nuclei are arranged at nearly equal distances, and frequently exhibit distinct nucleoli. Sometimes these fibres shew a disposition to split into very delicate fibrillæ. Acetic acid dissolves the fibre, leaving the nuclei unchanged. These fibres, containing nothing analogous to the white substance of Schwann, are devoid of that whiteness which characterises the tubular fibre; and it would seem that the gray colour of certain nerves depends chiefly upon the presence of a large proportion of the gelatinous fibres. Hence they are sometimes called *gray fibres*. The mode of connexion of the gelatinous fibres with the elements of the nervous centres is, as yet, quite unknown. They are found, in considerable numbers, in what are called the roots of the sympathetic, or the communications of that nerve with the spinal nerves; it has been supposed by Valentin that they are continuous with certain elements of the vesicular nervous matter.

"These fibres are smaller, in general, than the tubular fibres; their diameter ranges between the $\frac{1}{6000}$ and the $\frac{1}{4000}$ of an inch. They resemble very much the fibres of unstriped muscle." P. 212.

ORIGIN AND TERMINATION OF NERVES.—The exact relations of the central ends of the fibres on the one hand between themselves, and on the other with the grey vesicles or nucleated corpuscles, are not known. Allusion has been made above to the fibrous prolongations of the caudate vesicles, and further, it may be stated that the nerve-tubes are most intimately related to the nucleated corpuscles, adhering to and even indenting their sheaths; many of them also pass between these bodies, probably to form connexions with those which are more distant. It is conceived by Valentin that a looped disposition of the fibres takes place in the gray substance of the nervous centres; but Todd and Bowman state they have not been able to discover such an arrangement.

A just conception of the psychical phenomena, and especially of the relations existing between pure voluntary motion and the senses, would lead the physiologist to infer that no such connexion as that described by Valentin really exists, and for this simple reason, *that it is not wanted;* for it is certain, although the fact is not sufficiently kept in view, that there is no necessary connexion between voluntary motion and sensation. But in the case of the spinal cord all is reversed, for it is at length admitted on all hands that here impressions do pass, and necessarily, from the afferent to the efferent fibres, whatever these may be. It is our own fixed conclusion, not resting, however, on Stilling's dissections, but on what can be seen with the naked eye on carefully following the fibres of the anterior and posterior nerve-roots, that a part of their fibres unite in the gray substance; an opinion which, as will be presently shown, is supported by the dissections of Budge in the vertebrata, and by those of Newport in the case of the avertebrate animals.

Although the minute connexion of the two organic nervous elements are thus not precisely made out, it may, we think, be stated that, whatever these may be, *they are essentially uniform throughout the whole system*—in the convolutions of the brain, in the laminæ of the cerebellum, in the corpora striata, in the spinal cord and all the ganglia, whether spinal or sympathetic: this is the conclusion at which we had long since arrived, and recent research confirms its correctness.

As to the mode in which the nerve-tubes terminate, to use the conventional term still employed, it appears that it is always by *loops*, not by points or free ends; even in the olfactory, optic, and auditory nerves, where the latter disposition was formerly thought to obtain, the termination is by loops.

This point being established, it still remains to be determined whether the *end-loops*, as the German writers call them, are in all cases formed exclusively by tubes of a similar nature, that is in one case of sentient fibrils, in another case of motor fibrils; or whether, in one and the same loop a sentient and a motor fibril run into or join one another. As this is a question of considerable importance in the investigation of the laws of innervation, it may not be superfluous to enquire, what light can be thrown upon it either by inference or by direct anatomical observation. The only writer we are acquainted with who has fully entered upon the question is Valentin, who, in his earlier works, and again in his admirable *Lehrbuch der Physiologie*, has well illustrated the subject. He remarks, that if we assume, for the sake of argument, that "a sentient primitive fibril unites with a motor primitive fibril to form the loop, when the will is exerted to determine motion, it must happen (according to the laws of nervous conduction) that the stimulus descending in the centrifugal direction will cause the corresponding muscular fibres to contract, and will then stream back again, centripetally, and thereby produce a sensation," a condition of action which never takes place. "Thus the improbability of an occurrence like the one supposed, justifies us in rejecting such an hypothesis. But further, the anatomical disposition is also conclusive against such an arrangement: the existence of terminal loops in the retina, in the vestibule, and in the cochlea (and we may add in the papillæ of the skin), allows of no doubt that in these parts similar, that is sensible, fibrils alone unite. Again, there are numerous terminal plexuses and loops in very sensitive parts, which are yet incapable of any considerable motion; for example, however highly we may estimate the power of contractility in the fibres and vessels of the dental papillæ, still it can never be assumed that the one half of their nerves are subservient to this action, and only the other half to the production of sensation." (l. c. p. 595). We may, from these and similar facts, safely conclude that, in the case of sentient nerves, the terminal loops are formed exclusively of sentient fibrils. With respect to the motor nerves the anatomical evidence is not so complete, for, although the nerves going to muscles consist principally of the motor class, yet without a single exception, but in very varying degrees, they receive some of the sentient order likewise. It may, however, be inferred, so far as analogy and the laws of innervation justify an inference, that here, as in the purely sentient organs, the individual loops are formed either entirely of motor or entirely of sentient fibrils.

The general conclusion then is, that, both as regards the central spinal and peripheral parts of the system, but more certainly in the latter than in the former case, the primitive fibrils form loops, so that, if the cerebrum be excluded, it may be said, in the language of Dr. Carpenter, that "it appears uncertain from the results of the most recent microscopical inquiries, whether the nervous fibres can be said to have any distinct *terminations*, either in the gray centres, or in the organs to which they are distributed."

Comparative Anatomy.—In the lowest tribes of the animal kingdom, no satisfactory evidence of a nervous system has hitherto been discovered; but some of their actions appear to be of voluntary character, and as consciousness cannot be conceived to exist without the agency of some form of brain or nerves, the circumstance of these having as yet escaped detection cannot be regarded as a proof of their non-existence. It has been commonly supposed that, in the *Acrita*, the nervous system assumes a diffused or molecular form; but the mode in which this system operates seems to demand in all cases, as a necessary condition, the presence of an active centre and conducting fibres. The following passage from Dr. Carpenter's Physiology expresses what we have long urged as arguments against the more commonly received opinion.

"Moreover, some of their actions appear to show a certain degree of *voluntary* power, and therefore of consciousness; being independent, so far as can be ascertained, of the operation of external stimuli. These phenomena, then, would lead us to suspect the existence of a nervous system in the beings which exhibit them; not, however, in a '*diffused*' condition, but in the form of connected filaments. For, what consentaneousness of action can be looked for in a being, whose nervous matter is incorporated in the state of isolated globules with its tissues? How should an impression made on one part be propagated by these to a distance? And how can that consciousness and will, which are *one* in each individual, exist in so many unconnected particles? If, then, we allow any sensibility, consciousness, and voluntary power, to the beings of this group of Acrita—to deny which would be in effect to exclude them from the Animal Kingdom—we must regard these faculties as associated with nervous filaments, of such delicacy as to elude our means of research. When the general softness of the textures, and the laxity of structure that characterises the nervous fibres, in the lowest animals in which they *can* be traced, are kept in view, little difficulty need be felt in accounting for their apparent absence." P. 97.

The study of the comparative anatomy of the nervous system, when conducted upon philosophic principles affords, as in all other branches of organization, results of the highest interest. We are sorry that the impartial discharge of our duty will not allow us to speak in terms of commendation of the article upon this subject in the 25th part of that valuable work, the Cyclopædia of Anatomy and Physiology.

It does not, appear to us that Mr. Anderson has been happy either in his descriptive details or in his physiological deductions; and specially we miss those important generalizations, regarding both structure and function, which the advanced state of neurological knowledge would equally justify and demand.

The object of Mr. Anderson "is to describe the anatomy of the nervous system in the different classes of animals as they rise upward in the scale;" and after noticing the nervous ring placed around the mouth of the echinodermata, the writer thus defines the principle of progressive development."

"We may next ask what are the characteristics of an increase in development of the primary nervous ring just mentioned, as the fundamental form of every nervous system. They are precisely these: either that it is in itself more highly developed, or that it is multiplied and repeated several times. This we shall find illustrated in nature; the former in the Mollusca, the latter in the Articulata." P. 603.

In following out the details of this proposition, Mr. Anderson has confounded parts together, which are in reality perfectly distinct organs. In the common star-fish there is, as just stated, a ring, consisting of small swellings usually regarded as ganglia, which correspond in number to the rays; of threads, or commissures, uniting them together; and of nerves or conductors. Such being the elementary parts, we may be assured that, when they are developed in the higher animals, however much altered in form, size, and position, they still retain their original endowments. But the writer, if we rightly apprehend his meaning regarding the "primary nervous ring," considers that the kind of open ring contained in each segment of the articulate animals, "formed of a ganglion and two semi-circular radiating nerves," is a repetition of the nervous ring of the asterias, thus confounding together nerves and commissures; whilst, in another place, speaking of the vertebrata, he says "the primary nervous rings of the preceding (invertebrate) classes have become ganglions, and their commissures have become primary nervous rings," thus again confusing, if we seize the author's meaning in this somewhat obscure passage, organs of a totally different character, namely, nerves and commissures. And we would here remark, that the existing knowledge relating to the nervous systems of the echinodermata, is not sufficiently accurate to serve as the basis for comparison with the component parts of the same system in higher animals; but it is particularly desirable to point out that, if, which is most probably the case, the nervous ring of the asterias represents at once the cerebrum and spinal cord, it is impossible to compare that structure with a single segment either of the brain or of the spinal cord. But if any comparison is to be made, it is quite certain that each ganglion of the articulata and each segment of the spinal cord of the vertebrata, correspond to the gangliform swelling of the asterias—that the longitudinal commissural fibres of the spinal cord (assuming that direction in obedience to the altered form of the animal) correspond to the circular commissural fibres of the radiata, and that the nerves issuing from the spinal ganglia represent the nerves which, in the star-fish, run into the arms. We have pointed to these errors, because, if the relations of the several organs existing in different classes are not truly interpreted, comparative anatomy, instead of elucidating and explaining, which in reality it does in an eminent degree, the true nature of the involved and complex structures of the higher vertebrata and man, will only mislead and confuse.

The successive development, as traced by Mr. Garner in mollusks is most instructive, and proves, in a manner not to be mistaken, that the theory which attributes an independent power to every ganglionic mass has a real foundation in Nature. Mr. Garner thus expresses his views, in the truth of which we generally coincide.

"The arrangement of the nervous system in Conchifera is of the highest physiological interest. It affords a beautiful example of a complete analysis of the more complicated nervous system of the vertebrata. We have here an anterior pair of ganglia, from which filaments proceed to all parts of the body, associated too with the ingestive faculty; they are connected with whatever degree of psychical endowment the animal possesses and form its sensorium commune; they are the source of its voluntary actions. The respiratory organs likewise have their special centre in the branchial ganglion or ganglions, the development of which is always proportioned to that of the branchiæ. And there is a special centre provided for the locomotive organ, too, whose development is strictly in relation with its size and activity, and which is absent when that organ does not exist. And it must be observed that these special ganglia (respiratory and pedal), although unconnected with each other, communicate with the œsophageal ganglia.

"Have we not here distinctly marked out the cerebrum (the centre of volition and sensation), the medulla oblongata (the respiratory centre), and the cerebellum (locomotive centre), as they occur in the higher vertebrata? And in the aggregate of the chords by which the œsophageal ganglia communicate with the pedal and branchial ones, do we not see the analogue of at least a portion of the spinal cord, that portion which consists of afferent and efferent nerves to and from the brain? The nervous system is distinctly adapted to the wants of the animals and their limited psychical endowment, and the same law prevails throughout the scale of animals. It is not the nervous system which develops the powers and instincts of the animal; on the contrary, these latter determine the development of the nervous system. This is well illustrated by a comparison of the oyster and the mussel. These mollusks differ only in a greater locomotive power belonging to the mussel, to effect which it possesses an organ called the foot; the oyster is devoid of such an organ. The mussel has an additional ganglion (the pedal) which the oyster has not, and this ganglion is not an isolated centre, but, like the branchial ganglion, is connected by distinct filaments with the anterior or cerebral ganglia." P. 604.

Mr. Newport's researches on the nervous and vascular systems of the Myriapoda and Arachnida, constitute one of the most important additions to this interesting branch of anatomy that has appeared during some years. We noticed in our last number the disposition of the blood-vessels, the great trunks of which, and especially in the scorpion, have every appearance of being formed upon the same principles as those of the vertebrata. The existence of aortic arches in these avertebrate families is full of interest to the inquirer into embryology and comparative anatomy.

The principle upon which the gangliated cord of the articulata, and therefore of the spinal cord, for there can be no doubt that these really are, what Sir C. Bell pronounced them to be, corresponding organs, is highly instructive in relation to the laws which govern the nervous centres in all classes. It has long been known in a general way, that in the articulate type, the number of the spinal ganglia and of the pairs of nerves therefrom arising, is determined by the number of segments into which the body is divided; but the exact application of this rule is not so well understood as from its importance it deserves to be.

There are few classes in which this principle is better seen than in the *annelida*, and especially in the common lumbricus terrestris, in which, on close examination, a minute series of gangliform enlargements is seen, corresponding in number to the moveable segments into which the animal is divided. That each of these ganglia is a source of innervation, we ascertained some years ago by dividing several earth-worms, and observing that, as the rings of the caudal segment died and mortified day by day, (for it is a total fallacy to assert that the posterior part of these annelides, when cut off, becomes converted into a new and perfect creature, which, at the time we refer to, was a common opinion,) those which continued alive still retained the power, even till only two or three rings remained, of exciting motion when the skin was touched.

Mr. Newport has given other and instructive examples of this law. Thus he found, by carefully investigating the two great divisions of the Myriapoda—Chilopoda, and Chilognatha, that each of these segments, into which the body is divided, instead of being, as it appears, simple, is in reality double, and that each sub-segment contains its appropriate ganglion; a principle which also applies to the head, this being composed of several segments, four or six in number, each having its pair of cephalic ganglia. In the julus terrestris the division is carried so far that there are no less than 96 ganglia in its elongated cord. In many instances this typical formation can only be detected, as in so many analogous cases, by watching the progress of development; thus, in the embryo of *Necrophlœophagus longicornis*, on bursting the shell, has four double encephalic ganglia, the centres of a corresponding number of segments. But, in some instances, as in polydesmidæ, the ganglia of the sub-segments remain through life distinct in the posterior part of the body, whilst in the anterior, and especially in those nearest the head, they have coalesced, a species of metamorphosis which is familiar to those who are acquainted with the transformations occurring in the chrysalis among insects, and in the higher or decapod crustaceans, as the astacus fluviatilis. Thus in Myriapoda the encephalon is formed by the aggregation of several separate (at least four) pairs of ganglia placed above the œsophagus; but the interpretation of the physiological signification of these masses, a point of much importance, is most difficult of attainment. Mr. Newport, indeed, who regards the first pair as the cerebral lobes, conceives that the second pair of ganglia, which furnish the optic nerves, as the first pair do those of the antennæ, constitute, as in insects, the organs of volition; but it is most doubtful if any such subdivision of the true organ of consciousness ever occurs, and we believe that the only explanation which will be found to harmonize with the results of comparative anatomy, is, that in every class of animals, invertebrate as well as vertebrate, the most anterior pair of cephalic ganglia is the essential and exclusive seat both of sensation and volition.

One of the principal objects of these researches is to prove that, in the Myriapoda, as in insects, there is a set of nerves anatomically and physiologically distinct from the ordinary nerves of volition and sensation, and which Mr. Newport calls, though not very appropriately, "the fibres of re-inforcement of the cord." They enter into the formation of all nerves which spring from the gangliated or spinal cord, and are observed to form the borders of these nerves, and the outer part or sides of the spinal cord between them; and thus it is conceived by this physiologist, that "every nerve arising from a ganglionic enlargement of the cord is composed of four sets of fibres; an upper and an under one, which communicate with the cephalic ganglia; a transverse or commissural, that communicate only with corresponding nerves on the opposite side of the body; and a lateral set, that communicate only with nerves from a ganglionic enlargement on the same side of the body, and form part of the cord between the roots of the nerves." It is conceived by Mr. Newport that these last fibres constitute in reality an *excito-motory* system, independent of the *sensori-volitional;* and this view is supported by several experiments. Dr. Todd and Mr. Bowman do not agree in these deductions; and, among other objections, state that it would appear the above fibres merely pass from nerve to nerve without forming any junction with that which is doubtless the source of all the excito-motory power, the vesicular matter, namely, of the cord. It must be acknowledged that, neither in the description nor in the accompanying plates, is such a junction indicated by Mr. Newport; but we have distinctly seen, on examining aquatic larvæ, and especially those of some species of gnat, that the fibres of re-inforcement *do form a*

*connexion with the granular matter of the ganglia*, and we think that what happens in the case of insects may be safely applied to that of the myriapoda. On the whole, this discovery of this additional set of nerves must be regarded as an important step gained towards the solution of that most difficult problem, the true anatomy of the spinal cord, and the mode and origin of the spinal nerves.

The observations of Dr. Will relate to the minute structure of the ganglions and to the origin of the nerves in avertebrate animals. His account of what should be regarded as the neuro-skeleton, in the crab and gasteropod mollusks, where it consists of a peculiar cellular membrane, is interesting when contrasted with the same structure in the myxinoid fishes, and especially in the amphioxus, in which remarkable animal it is equally of a membranous consistence; links like these, to which should be added the commencing cartilaginous cranium of the cephalopoda, serve to unite the vertebrate with the invertebrate classes, and so to obviate the seeming void by which it has been supposed they are separated. In fact, for ourselves, we have no hesitation in admitting, in all animals having a distinct nervous system, the two typical forms of skeleton expressed by the terms neural and dermal skeletons. In some classes, as insects, crustaceans, &c., the former is reduced to the membranous form, whilst the latter is much developed, consisting of a horney, pergamentaceous, or calcareous envelope; whilst in others, as the vertebrata, the neural skeleton is developed at the expense of the dermal, which re-appears, however, in many classes, as in the scales of fishes, especially of the ganoid type, in the saurian reptiles, and more palpably still in several among the existing and extinct edentata, as the armadillo and glyptodon. A minute account is given of the connexion of the nerves, but which, after our notice of Mr. Newport's researches, need not detain us.

According to Dr. Todd and Mr. Bowman, although in all essential points the structural arrangement of the nerves and nervous centres of the invertebrate classes corresponds with that of the vertebrata, yet there are some differences, which are thus described.

"In the lobster, the nerve-tubes are large; the tubular membrane has the same transparent, homogeneous appearance, which we have noticed in the vertebrata. But it incloses many delicate nuclei at various intervals. Within the tubular membrane there is a very thin layer of the white substance of Schwann. The nerve-tubes are very transparent, and are much larger than the average size in vertebrata. Respecting the existence or structure of the gelatinous fibres, we can offer no remark. In insects and myriapoda, the nerve-tubes vary considerably in size; they are collected into bundles, and are surrounded by a transparent sheath of homogeneous membrane, which accompanies the larger ramifications of the nerve-trunks. The white substance of Schwann is not so obvious nor so constant in these nerves as in those of the lobster, and the existence of nuclei makes them resemble closely the gelatinous fibres of the vertebrata. The anatomical characters of the vesicular nervous matter of invertebrata do not essentially differ from those of the same substance in the vertebrate classes, so far as our observation enables us to judge. The nerve-vesicles with nuclei and nucleoli are equally apparent in both, though in the former they are more transparent, and contain less pigment." P. 227.

A singular little fish, the Amphioxus Lanceolatus, has lately attracted the attention of naturalists, and of which a minute account has been published by Mr. John Goodsir. The vertebral formation is reduced to its simplest condition, consisting of a chorda dorsalis formed externally of a fibrous sheath, and internally of an immense number of laminæ of the consistence of parchment, and representing so many individual vertebræ. The most extraordinary circumstance, however, is that, according to Mr. Goodsir, the animal has neither cranium nor brain, though Retzius admits the latter. The nervous system is thus described.

"The spinal cord is situated on the upper surface of the chorda dorsalis, enclosed in the canal formed in the manner above described. When the whole length of this canal is displayed by removing the muscles, and then carefully opened, the spinal cord is seen lying in the interior, with nerves passing out from it on each side. It stretches along the whole length of the spine, is acuminated at both ends, and exhibits not the slightest trace of cerebral development."

"From fifty-five to sixty nerves pass off from each side of the cord; but, as the anterior and posterior vertebræ are very minute, and run into one another, and as the spinal cord itself almost disappears at the two extremities, it is impossible to ascertain the exact number, either of vertebræ or of spinal nerves. These nerves are not connected to the spinal marrow by double roots, but are inserted at once into its edges in the form of simple cords."

"When an entire animal is examined by transmitted light, and a sufficient magnifying power, the anterior extremity of the spinal cord is observed, as before mentioned, to terminate in a minute filament above the anterior extremity of the vertebral column. The first pair of nerves is excessively minute, and passes into the membranous parts at the anterior superior angle of the mouth."

"One of the most remarkable peculiarities in the Lancelet is the absence of the brain. Retzius, indeed, describes the spinal marrow as terminating considerably behind the anterior extremity of the chorda dorsalis, in a brain which exhibits scarcely any dilatation; but careful examination of the dissection of my own specimen, which I have also submitted to the inspection of Dr. John Reid. and of other competent judges, has convinced me that the spinal cord, which may be traced with the greatest ease to within 1-16th of an inch of the extremity of the chorda dorsalis, does not dilate into a brain at all."

"The peculiarities in the structure of the spinal cord are not less remarkable than those of its configuration. It is difficult to understand, according to the received opinions on the subject, how a spinal cord destitute of primitive fibres or tubes, and composed altogether of isolated cells, arranged in a linear direction only towards the middle of the cord, can transmit influences in any given direction; and more especially how the tract of black or grey matter, if it exercises any peculiar function (excito-motory) communicates with the origin of the nerves. The nerves, also, are remarkable, originating in single roots, and containing in their composition one kind only of primitive fibres (cylindrical)." P. 618.

We must refer to the original paper in the Transactions of the Royal Society of Edinburgh, or to the copious extracts contained in Dr. Todd's Cyclopædia, for a more detailed account of this apparently anomalous member of the vertebrate family.

Dr. Budge, who is known as the author of several contributions to the nervous system, has communicated, in Müller's Archives for the last year, a very minute, and apparently correct, account of the mode of origin of the spinal nerves in the frog.

After noticing the opinions of Gall, Keuffel, Bellingeri, Weber, and Valentin, all of whom admit the connexion between the roots of the nerves and the gray substance of the cord, Dr. Budge particularly criticises and opposes the views of Stilling and Wallach, according to whom all the fibres of the nerves pass transversely through the gray matter. The dissection of the lower extremity of the cord or *conus terminalis*, proves, according to

Budge, the interesting fact, that "many of its longitudinal fibres are the continuation of the roots of the nerves which enter the cone."

At the point of junction the fibres of the nerves experience a change of colour from blueish gray to yellowish gray, and also of size, for whilst their diameter equals $\frac{1}{250}$ of a line, the longitudinal fibres of the cone are only $\frac{1}{400}$ to $\frac{1}{800}$. He has followed many of the longitudinal fibres from their origin out of the nerves as far as to the anterior end of the conus, and from this into the longitudinal fibres of the spinal cord itself.

The examination of the nerves higher up, gives results to which we attribute much importance. If one of the roots, the dorsal for example, be traced, it is found to divide into two fasciculi; one of these, the more superficial, forms a slight curve, and then ascends towards the brain, forming, with similar fasciculi of other roots, the posterior column or strand of the spinal cord. Dr. Budge remarks, "it is certain that the above course of the fibres is natural and not artificially made by rupture and displacement arising from compression. If the above-named white band (the posterior column) be cut off with the nerve-root attached in a perfectly recent specimen, although some fibres are torn many remain, and thus the entrance of an individual fibre of the nerve-root into a medullary tube of the spinal cord may be observed." We may remark that Valentin had already represented this connexion between the nerve-tubes and the varicose fibres of the cord. As regards the deeper-seated fasciculus, Dr. Budge found more difficulty in following the fibres; he ascertained, however, that they penetrate into the gray substance, and here, being spread out into three or four bundles, and separated by the ganglionic corpuscles, he concludes that they, like the more superficial ones, turn upwards towards the brain.

An examination of the plates accompanying this description induces us to believe that many of the last-named fibres terminate in the gray substance; the author indeed himself found a number of fibrils uniting the corpuscles of that substance together like bridges, and thinks some of them may be derived from the nerves. We may observe that the greatest care was taken to obviate error, and Dr. Budge particularly avoided the use of the compressorium, to which, by causing rupture and displacement of the fibres, he attributes what he considers to be the mistakes of Stilling respecting the transverse course of the nervous fibres within the cord. As we shall have occasion again to refer to these dissections, it will suffice to state here that they seem to establish two positions:—

1. That each root of the spinal nerve divides into two fasciculi.
2. That the fibres of one of these fasciculi become directly continuous with the white fibres of the cord; and the fibres of the other fasciculus with the gray substance of that body.

We shall conclude our present notice by a brief account of two remarkable forms of nervous organs—the peculiar nervous system of the Torpedo and the Pacinian Corpuscles. Appended to the highly important work of Matteucci on Electro-Physiological Phenomena, of which an account was given in the last Number of this Journal (see Medico-Chirurgical Review for April, p. 305), is a very interesting account of the nervous system and electrical organ of the torpedo by M. Paul Savi, a colleague of Matteucci. It is well known that this remarkable animal, of which, besides many others existing in the seas of warmer climates, there are three species in the Mediterranean, has a large electrical organ situated on either side between the branchiæ and the heart. As our limits will not allow us to follow M. Savi into the details he has given respecting this most interesting apparatus, we must restrict our notice to the nervous structures by which it is supplied.

These consist of 1, a great branch of the 5th pair; 2, three very large branches of the 8th pair; 3, a small branch of the 8th. In order to understand the relations of these nervous cords to each other, it is requisite to describe shortly certain peculiar masses which are developed in the encephalon of the electric ray, in addition to the parts existing in the *raia batis* and other species. The cerebral masses consist of the cerebral hemispheres or first encephalic mass; of the two bodies which, according to Straus, correspond more particularly to the superior quadrigemina or nates; and of the third encephalic mass of Carus, or the cerebellum; of the medulla oblongata; and of two masses, each divisible into two lobes, which are called, the most anterior, *restiform laminæ* by Serres, whilst the two posterior are called by Savi the *electric lobes*. The two former, or restiform laminæ, constitute two peculiar lateral lobes to the cerebellum; they present a very decided convoluted disposition; consist of a process of white substance; are united by fibres to the electric lobe immediately to be described, and give origin to the principal part of the superior root of the 5th pair, which goes to be distributed almost entirely to the muciparous organs, which are greatly developed in the torpedo.

Savi says, " the most important part of the brain in this animal is unquestionably the medulla oblongata, on account of its presenting a development superior to that which it possesses in non-electric fishes; and, still more, because it is composed of certain lobes which have an intimate relation with the nerves of the electric organs. It has half the length of the entire encephalon and is broader than any of other parts of the brain." The great peculiarity consists of two large oval masses, larger than any of the others, which entirely fill the rhomboidal sinus of the fourth ventricle, called by Savi, who considers them to represent the posterior pyramids of the medulla oblongata of other animals, the *electric lobes*. They were first noticed by Jacopi, and are thus described in his Elements of Physiology and Comparative Anatomy, published in 1810:—" behind the cerebellum, and precisely in the place where in other fishes the encephalon becomes continuous with the medulla oblongata, there is in the torpedo a swelling of gray substance, of which the volume is almost larger than that of the cerebellum and cerebral lobes together. It is from this swelling that arise on each side, three large nervous trunks (those of vagus) which are distributed, in great part, to the electrical organs." There are thus in the torpedo two distinct masses—the restiform appendages of the cerebellum and the electric lobes, which do not exist in common rays; a fact than which it is impossible to conceive any thing more corroborative of the opinion now become prevalent, that the gray substance is the source of all power in the nervous system.

The account of the origin of the 5th and 8th nerves is interesting: that part of the former, the upper, already alluded to as being attached to the lateral mass of the cerebellum, does not actually arise there, but can be traced into the electric lobe, which also gives origin to the 8th, so that the two nerves engaged in supplying the electric apparatus arise from the same mass, and that a body which is peculiar to the torpedo. M. Savi succeeded in tracing the vagus into the gray substance, and he concludes from his observations, that the elementary fibres of that nerve, as well as of the 5th, form loops within the electrical lobe; but he does not appear to have shown that the fibrils of the two nerves join together. In connexion with this subject, we may state that Stilling and Wallach have traced the vagus to a nucleus of gray substance on the floor of the 4th ventricle in mammals.

The most original and important part of M. Savi's Memoir is that which relates to the minute structure of the electrical organ, and especially of its nerves. The first minute account of this curious apparatus was given by Hunter; he states that the columns forming are very numerous, varying according to the size of the animal; in one specimen 470 were counted. Each column is divided horizontally by a vast numher of diaphragms into small cells, which contain a glairy fluid. M. Savi thinks each partition is composed of two very thin layers, and that, in fact, " each prism or column of the organ is formed of a great number of membranous cells superposed one upon the other, almost soldered as it were together, and enclosing between the faces which touch the sanguiferous vessels and the elementary nervous fibres." Some estimate of the complexity of this instrument may be formed from the fact stated by Hunter, that, in a column one inch long, there were as many as 150 septa.

The proper nerves of the apparatus are derived exclusively from four great branches of the vagus: the primary tubules present the double contour, and what is interesting, whilst the other and ordinary branches of the 8th, those of the stomach, and the one called by anatomists nervus lateralis, present gangliform enlargements, those going to the organ are entirely devoid of them. The nervous branches penetrate between the prisms, and then send off very minute twigs, in fact primary tubules, into each diaphragm or septum, where, and apparently between the two layers above described, they form a very delicate network, the meshes being, although occasionally irregular, usually octagonal in figure. A high magnifying power and much care in the manipulation are required to display this distribution. The septa, as Hunter had already seen, are very vascular.

It is impossible in considering this interesting formation to overlook its marked resemblance to the galvanic battery, for, as Matteucci has experimentally ascertained, a very small portion of a prism gives an electrical discharge, corresponding to the size of the section, upon irritating its nervous filament; it is further certain that, like single pairs of plates, each portion can develop electricity. We may likewise remark, that Galvani obtained a feeble spark from the torpedo, and more lately M. Matteucci: Dr. Davy has also also shown that needles may be rendered magnetic by the discharge.

M. Savi has discovered a very curious apparatus in the torpedo which is alike distinct from the electrical organ, and from those numerous muciparous follicles which all the rays and sharks possess; he calls it the "*follicular nervous apparatus*." It is found on the border of the mouth and nostrils, extending upon the periphery of the electric organs; and it consists of a vast number of follicles placed in rows, each composed of two membranes, and supported by a fibrous structure. Each follicle receives a twig of the peculiar branch of the fifth pair already mentioned, which, after forming a small swelling, enters the little sac, and then again leaves it diminished in size to unite with the filament of the adjoining follicle. The use of this remarkable and highly-organized apparatus, which was first described by M. Savi at the scientific meeting held at Florence in 1840, is not known: but as it is confined to the torpedo, and is supplied by a branch of the fifth, peculiar to these fishes, proceeding from the electrical lobe of the medulla oblongata, there is great reason to conclude that this nervous follicular structure is in some manner or other connected with the development of the electrical power. The description given by M. Savi of the parts we have noticed, is accompanied by some beautifully executed and demonstrative engravings.

Pacinian Corpuscles.—These curious bodies are found in great numbers in connexion with the nerves of the hand and foot; more sparingly on other spinal nerves, and on the plexuses of the sympathetic, but never on the nerves of motion. They were discovered by Pacini in 1830; lately, Henle and Kölliker have given a very minute account of their anatomy, and they have again been examined by Dr. Todd and Mr. Bowman, and from their observations, which coincide with those of the German anatomists, the following details are extracted.

"They always present a *proximal end*, attached to the nerve by a *stalk* of fibrous tissue, prolonged from the neurilemma, and occasionally $\frac{1}{10}$ of an inch long; and a *distal end*, lying free in the areolar tissue. The corpuscles in the human subject have an average length of from $\frac{1}{20}$ to $\frac{1}{10}$ of an inch.

"A minute examination of these singular bodies discloses an internal structure of a highly interesting kind. They consist, first, of a series of membranous capsules, from thirty to sixty or more in number, enclosed one within the other; and, secondly, of a single nervous fibre, of the tubular kind, enclosed in the stalk, and advancing to the central capsule, which it traverses from end to end.

"Ten or fifteen capsules may be observed to be in contact with one another, while the rest are separated by a clear space containing fluid."

"There are generally a few capillary blood-vessels ranging over the surface of the corpuscles; but the capsules are chiefly supplied by a minute artery that enters in the fibrous tissue of the stalk, sends off a few capillaries which perforate the tubular canal, and form each a short loop in the intercapsular spaces." 398.

The use of the Pacinian bodies is at present quite unknown; but it has been surmised that they may influence the contained nerve and thus those with which it is connected, or "the apparatus may be the special instrument of some peculiar vital agency, which the nervous filament is designed simply to bring into communication with the nervous system." This latter view corresponds with the comparison which Pacini had himself already made between the structure of these bodies and that of the electrical organs of the Torpedo.

In our next number we purpose to consider the works, the titles of which are appended to the present article, in reference to Human Anatomy and also to the Physiology of the Nervous System.

